# “Chocolate” Gold Nanoparticles—One Pot Synthesis and Biocompatibility

**DOI:** 10.3390/nano8070496

**Published:** 2018-07-05

**Authors:** Neelika Roy Chowdhury, Allison J. Cowin, Peter Zilm, Krasimir Vasilev

**Affiliations:** 1 School of Engineering, University of South Australia, Mawson Lakes SA 5095, Australia; neelika.roy_chowdhury@mymail.unisa.edu.au; 2 Future Industries Institute, University of South Australia, Mawson Lakes SA 5095, Australia; allison.cowin@unisa.edu.au; 3 Microbiology Laboratory, Adelaide Dental School, The University of Adelaide, Adelaide SA 5005, Australia; peter.zilm@adelaide.edu.au

**Keywords:** green synthesis, cacao, non-cytotoxic

## Abstract

The chemical synthesis of nanoparticles can involve and generate toxic materials. Here, we present for the first time, a one pot direct route to synthesize gold nanoparticles (AuNPs) using natural cacao extract as both a reducing and stabilizing agent. The nanoparticles were characterized by UV-visible spectroscopy (UV-VIS), dynamic light scattering (DLS), and transmission electron microscopy (TEM); and have excellent biocompatibility with human primary dermal fibroblasts.

## 1. Introduction

For decades, nanoparticles (NP) of noble metals such as gold, silver, and platinum have captivated the researchers and the general public with their remarkable physical and chemical properties, as well as for their potent therapeutic power [1]. Gold is one of the least reactive among the noble metals [2], but its nanoparticulate forms possess unique chemical, electrical, and optical properties [3]. These properties, which are size and shape dependent, can be tuned by a variety of means, such as the synthesis route, reactants, and experimental conditions [4]. Numerous applications have benefited from the special properties of gold nanoparticles, including optics, imaging, sensing, catalysis [5,6] and biomedicine [7,8] (in particular dentistry, cancer diagnostics, and photothermal and photodynamic therapies) [9,10,11].

As a result of the growing interest in gold nanoparticles, numerous chemical and physical synthesis routes have been proposed during the last decades [12,13]. However, major drawbacks with some of the conventional synthesis methods include toxic, hazardous chemicals and challenging reaction parameters [14,15,16]. Modern synthetic trends are shifting to alternative synthetic routes to minimize the use of harmful chemicals. Several studies have reported the benefits of the biosynthesis approaches using plant extracts, and unicellular and multicellular organisms [14,17]. Although chemically complex, phytochemicals have major advantages over other biosynthesis methods, as they are generally non-toxic to mammalian cell types and to the environment [14,18]. The use of plant derivatives also reduces the possibility for the absorption of toxic chemicals on the surface of the AuNPs [19]. Various studies have demonstrated the power of phytochemicals in gold nanoparticles (AuNPs) synthesis as well as the biocompatibility of the generated AuNPs to different cell types [14,17].

Here, we report for the first time, on the potential of cacao extract as a reducing and stabilizing agent in the synthesis of AuNPs. In addition to being a popular constituent in various foods and beverages, cacao has been speculated to alleviate health disparities such as aging, inflammation, depression, cancer, and stress [20,21,22,23,24]. The hypothesis behind this work is that oxalic acid, which is a constituent of cacao, can reduce Au^3+^ in HAuCl_4_ to metallic gold and stabilize the resultant nanoparticle colloidal solution. This hypotheses is also further substantiated by our previous work, where we reported the synthesis of silver nanoparticles (AgNPs) facilitated by cacao extract [25]. Herein, we extend this synthesis approach to prepare biocompatible ‘green’ gold nanoparticles and explore their properties. This easy single-step synthesis route was optimized and the prepared samples were characterized using UV-visible spectroscopy (UV-VIS), dynamic light scattering (DLS), and transmission electron microscopy (TEM). Finally, primary human dermal fibroblast (HDFs) cells were used to evaluate the biocompatibility of the gold nanoparticles. 

## 2. Materials and Methods

### 2.1. Reagents and Chemicals

Hydrogen tetrachloroaurate (HAuCl_4_), penicillin, streptomycin, and L-glutamine were bought from Pro Sci Tech, Kirwan, Australia. Cold pressed cacao powder was obtained from Forest Super Foods, Melbourne, Australia, and stored in an air-tight container (Goodguys, Adelaide, Australia). NaOH pellets, phosphate buffer saline (PBS) tablets, foetal bovine serum (FBS), nitric acid (70%), and Dulbecco’s Modified Eagle Medium (DMEM) were purchased from Sigma-Aldrich, Sydney, Australia. Hydrochloric acid (36%) was procured from Ajax Finechem Pty. Ltd., Sydney, Australia. All of the reagents were used as received. Ultra-pure MilliQ water (resistivity 18.2 Ω, Sigma-Aldrich, Sydney, Australia) was used for all of the experimental and cleaning procedures.

### 2.2. Synthesis of Gold Nanoparticles

The aqueous extracts of cacao were prepared by mixing a varying amount of cacao powder (Table 1) in 10 mL of ultrapure water (MilliQ system, Millipore Corp., Burlington, MA, USA) at room temperature. The extract obtained after the filtration (0.45 µm—sterile EO, Sartorius Stedim Australia Pty. Ltd., Dandenong South, Australia) of the suspension was stored for the synthesis of AuNPs. Then, the cacao extracts were mixed with aqueous solution of gold chloride (0.1 mg/mL in MilliQ water) (Table 1). The reaction mixtures were stirred continuously for 30 min at 100 °C under reflux. After 30 min, the heating source was removed, the reaction mixtures were cooled down to room temperature (25 °C), and stirring continued for 24 h. The periodic (30 min, 1 h, 2 h, 3 h, 4 h, and 24 h) monitoring of the prepared AuNPs was carried out using a UV-VIS spectrophotometer. The samples, S1, S2, S3, S4, and S5, refer to the AuNPs suspensions synthesized with 0.1, 1, 2.5, 10, and 50 mg/mL of cacao extract, respectively. The pH of the nanoparticle solutions was six.

### 2.3. Characterization

The progress of the reaction was periodically monitored using a Cary 5 UV-VIS spectrophotometer (Varian Australia Pty. Ltd., Mulgrave, Australia) at room temperature in the wavelength range of 400–800 nm. All of AuNPs’ suspensions were diluted 2X (*v*/*v*) with MilliQ water prior to UV-VIS spectral characterization. MilliQ water was used as a blank throughout the experiment. Quartz cuvettes were used for all of the measurements.

All of the samples were diluted to a suitable concentration using MilliQ water prior to DLS analysis to determine the hydrodynamic diameter of the nanoparticles. A Nicomp 380 particle size analyzer (Nicomp Particle Sizing Systems, Port Richey, FL, USA) operating at 25 °C was used for all of the DLS and zeta potential measurements. The mean hydrodynamic diameters reported are the average of the three measurements taken of the three independent nanoparticle batches (separate syntheses). Disposable plastic cuvettes were used for all of the measurements. All of the analyses were carried out at pH-6.

A ‘JEOL 2100F’ (Tokyo, Japan) transmission electron microscope (TEM), operated at an acceleration voltage of 200 kV, was used to determine the size and morphology of the synthesized cacao-AuNPs. Samples for the TEM analysis were prepared by depositing a small volume (10 µL) of the AuNPs solution on a carbon coated copper grid (ProSciTech, Kirwan, Australia). The grid was left to dry overnight at room temperature prior to TEM analysis. The crystal structure of the AuNPs were determined with the selected area electron diffraction (SAED) pattern obtained from TEM images.

### 2.4. Fibroblasts Study

Primary derived HDFs were gifted from Dr. Louise Smith, the University of South Australia. The HDFs were harvested and grown, as described elsewhere [26]. Briefly, cells were grown from frozen stocks and maintained in DMEM at 37 °C in 95% humidity and 5% CO_2_. The DMEM was changed every 3–4 days. Ethics approval was approved by the Ethical Committee at the Queen Elizabeth Hospital and the University of South Australia Human Ethics Committee, described elsewhere [26]. 

The viability of the cacao-AuNPs treated primary human dermal fibroblast (HDFs) cells was tested using a resazurin assay based on the reduction of non-fluorescent resazurin by metabolically active living cells to form fluorescent resorufin which was quantified using a microplate reader. Cells (1 × 10^4^ cells per well in DMEM) were seeded in 24 well plates on air plasma cleaned (5 min, 40 W, 2 × 10^−1^ mbar) thermanox coverslips and incubated (24 h in 95% air, 5% CO_2_ at 37 °C) until they reached 50 and 80% confluency. The DMEM was supplemented with FBS, penicillin (100 IU), and streptomycin (100 lg) (Invitrogen). After incubation, the DMEM was removed and the cells (50 and 80% confluent) were briefly washed with PBS. The cells were then treated with different concentrations of AuNPs (containing 500, 250, and 125 µg/mL of Au) prepared in warm DMEM and incubated for 24 and 72 h. After each incubation period, the media was aspirated again and the cells were rinsed with PBS. A stock solution of 110 mg/mL resazurin was prepared in phosphate buffered saline and filter sterilized using a 0.2 mm filter. The stock was then diluted 1:10 in fresh warm DMEM and 600 µL of the diluted solution was added to each well. After 1 h, 200 µL of the reduced solution was transferred into a 96 well plate and the fluorescent intensity was recorded using a plate reader (λ_ex_ = 544 nm and λ_em_ = 590 nm). Fresh DMEM without any AuNPs served as the control. The media from the wells were replaced with fresh DMEM every 2–3 days during the course of the assay. The percentage of cell viability was calculated with the following equation.

Viability (%) = 100 × Absorption_test_/Absorption_control_

### 2.5. Statistical Analysis

All of the statistical analyses were performed using graph pad prism 6 software. All of the data were expressed as mean ± standard error mean (SEM). Statistical significance was determined using one-way ANOVA with a Dunnett’s post-test. All of the experiments were performed in biological and technical triplicates on three separate days.

## 3. Results and Discussion

### 3.1. Synthesis and Characterization of AuNPs

**Figure 1:**
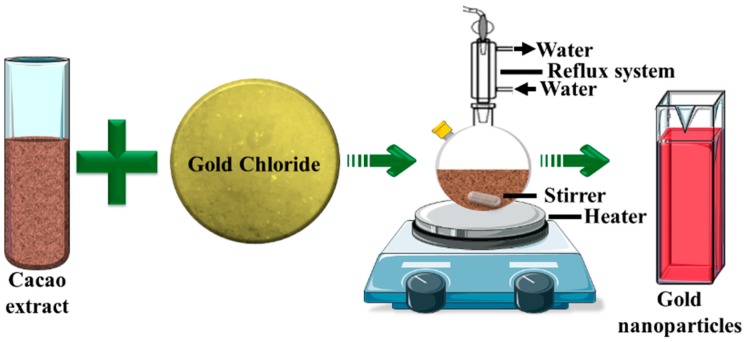
Schematic illustration of gold nanoparticles (AuNPs) synthesis from cacao extract.

**Figure 2:**
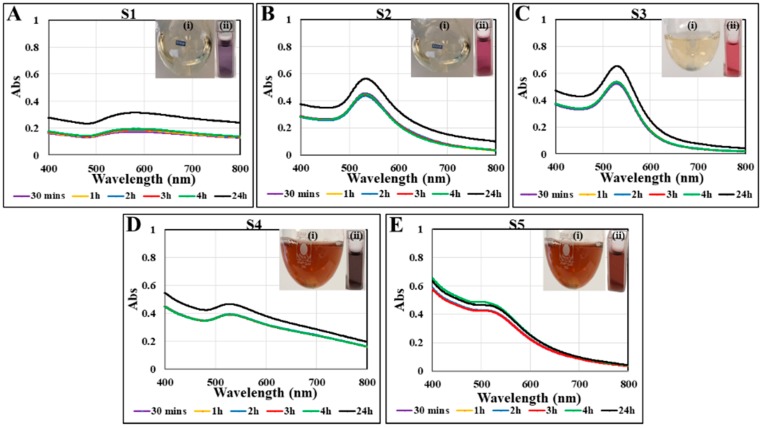
UV-Vis spectra of gold nanoparticles S1, (**A**); S2 (**B**); S3 (**C**); S4 (**D**); and S5 (**E**) obtained using cacao extract. Absorption spectra of samples recorded at 30 min, 1, 2, 3, 4, and 24 h from the initiation of AuNPs synthesis at 100 °C. Insets displaying the AuNPs solution before (i) and after (ii) synthesis.

The procedure for synthesizing gold nanoparticles with cacao extract is outlined in Figure 1. Briefly, under rigorous stirring, cacao extract was mixed with an aqueous solution of gold chloride (HAuCl_4_) at 100 °C (boiling temperature). Upon the addition of the reactants, the solution became increasingly darker and changed from transparent light yellow to purple-red within 5 min. The color change was a visual indication of the formation of AuNPs (Figure 2A–E insets). The samples, S1, S2, S3, S4, and S5, refer to the AuNPs suspensions synthesized with increasing the concentration of the cacao extract by 0.5, 1, 2.5, 10, and 50 mg/mL, respectively (see Table 1).

The intense purple-red color of the reaction mixture is due to the well-known phenomenon of plasmon resonance (PR), which is the result of resonant oscillations of the semiconfined electrons in the nanoparticles with the incident photons [27]. Several distinct parameters, including amount, size, and shape of the nanoparticles, interparticle electronic interactions, and the surrounding media have an influence on the position, shape, and intensity of the PR band [28,29]. UV-visible spectrophotometry was employed to evaluate the time course of the reaction kinetics by taking measurements at intervals of 30 min, 1, 2, 3, 4, and 24 h. UV-visible profiles of the reaction mixture at these time points are shown in Figure 2. The spectra of all of these samples exhibited a maximum of absorption (λ_max_) at around 530 nm, consistent with the plasmon resonance absorption band of AuNPs at ~510–560 nm [30], which confirmed the formation of gold nanoparticles. As an overall trend, the reaction had a fast-initial phase, much of the reduction being completed within 30 min. Clearly, there is no significant change in the absorption intensity within time points of 30 min, 1, 2, 3, and 4 h. However, a noticeable increase was observed after 24 h, which demonstrates that the reaction was slowly continuing, but was completed within this period as there was no further changes in the adsorption intensity beyond 24 h. 

The UV-visible spectra pointed to some interesting trends. Initially, when the cacao concentration increased to 2.5 mg/mL (S3) (Figure 2A–C), the intensity of the plasmon resonance absorption increased, which suggests the formation of more gold nanoparticles. In the same time, the maximum of the peak shifted to the left, indicating a decrease in nanoparticles size. A further increase in the cacao concentration to 10 and 50 mg/mL (S4 and S5, Figure 2D,E) led to a decrease in the plasmon resonance absorption and a broadening of the spectra, pointing to aggregations in the system. 

Oxalic acid in cacao is a natural reducing agent, which we showed to reduce silver ions into nanoparticles [25,31]. Oxalic acid exists as oxalate ions (C_2_O_4_^2−^) in the experimental conditions (pH > 4.3) used for this study. The standard potential of the C_2_O_4_^2−^/CO_2_ oxido-reduction couple is E^0^_red_ = −0.49 V, while the one of AuCl_4_^−^/Au(s) is E^0^_red_ = 1.002 V. The oxido-reduction reaction scheme resulting from the reduction of gold ions by oxalate is as follows:

C_2_O_4_^2−^ ⇌ 2CO_2_ + 2e^−^ AuCl_4_^−^ + 3e^−^ ⇌ Au_(s)_ + 4Cl^−^ 2AuCl_4_^−^ + 3C_2_O_4_^2−^ → 2Au_(s)_ + 6CO_2_ + 8Cl^−^ (1)

The synthesized AuNPs were characterized for their hydrodynamic diameter and zeta potential. The results are summarized in Table 2. The hydrodynamic diameter (as determined by the DLS) of the samples S1, S2, and S3 was 54, 29, and 18 nm, respectively. These results are in good agreement with the UV-vis absorption spectra, where the PR maximum shifted to shorter wavelengths, indicating a smaller particle size. The hydrodynamic diameter of samples S4 and S5 could not be reliably determined because of aggregates, also suggested by the UV-vis spectra. 

The zeta potential (ζ) provides important cues about the stabilization mechanisms in the colloidal suspension of nanoparticles. The zeta potential (Table 2) of samples S1, S2, and S3 was between −11 mV to −17 mV, and this range is known to confer incipient stability for colloids [32]. The negative charge on the surface of the nanoparticles appears to play a significant role by ensuring repulsion between the particles in the suspension. The samples were very stable and even after a month, no visible particle agglomeration was observed. 

**Figure 3:**
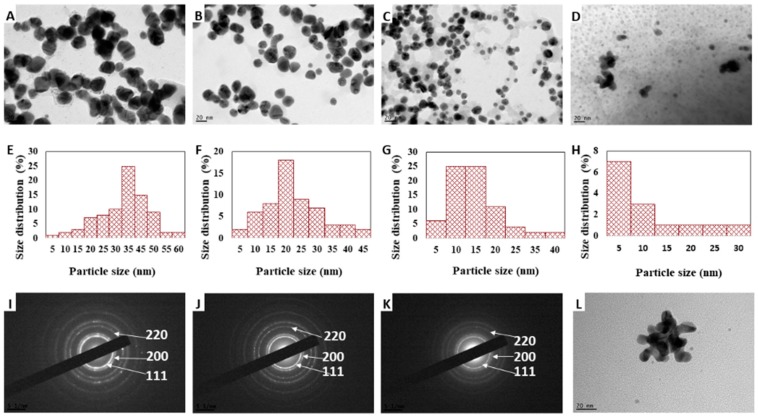
TEM images of the cacao-AuNPs S1 (**A**), S2 (**B**), S3 (**C**), S4 (**D**), and S5 (**L**). The corresponding histograms showing the particle size distribution (**E**, **F**, **G,** and **H** for S1, S2, S3, and S4, respectively). Representative SAED patterns of S1 (**I**), S2 (**J**), and S3 (**K**).

The TEM images in Figure 3 shows that the morphology of the as-synthesized AuNPs was mostly spherical, and there was no particle aggregation when the cacao concentration was below 10 mg/mL (Figure 3A–C). The particle size analysis of samples S1, S2, S3, and S4 are shown in Figure 3E–H, respectively. The AuNPs had an average particle size of 35 ± 10 nm (S1), 20 ± 9.1 nm (S2), 10 ± 11.6 nm (S3), and 7 ± 4.2 nm (S4). The particle sizes of samples S1, S2, and S3, determined from the TEM images, are smaller than the hydrodynamic diameter measured by DLS. These variations in the particles sizes are the result of the different measurement principles used by these two methods [33]. The size distribution analysis of sample S5 could not be performed reliably because of the presence of particle aggregation. However, both methods suggest an overall trend of particle size reduction when increasing the concentration of cacao was observed.

The selected area electron diffraction (SAED) analysis confirmed that the synthesized AuNPs are crystalline in nature (Figure 3I–K). As a result of random orientation of crystal planes, concentric diffraction rings were observed in the SAED patterns of the samples S1, S2, and S3, and the reflection rings were indexed to (111), (200), and (220) planes of the face centered cubic (fcc) crystalline lattice of gold.

### 3.2. Viability of Human Dermal Fibroblasts after Exposure to AuNPs

Gold nanoparticles have found numerous applications in advanced medical therapies ranging from the sensing to treatment of cancers. It is thus important to evaluate the cytotoxicity of this new nanomaterial to human cells. Human dermal fibroblasts were selected for this experiment because these cells play important role in connective tissue. We tested AuNPs resulting from S1, S2, and S3 only since these preparations were free of aggregations, which is important for potential applications.

Cells having two different levels of confluence (50% and 80%) were assessed for their viability after 24 and 72 h of exposure to concentrations of cacao-AuNPs that contained 125, 250, and 500 µg/mL of Au. Representative microphotographs of the untreated and HDFs treated with AuNPs are shown in Figure S1. After 24 h of exposure, 50% confluent HDFs showed no morphological changes in any treatment groups compared with the control (Figure S1A). At a treatment time of 72 h (Figure S1B), the cells morphology is similar to the control for all three of the AuNPs exposure concentrations. No cell shrinkage or floating cells were observed in the AuNPs treated HDFs at both time points. For the 80% confluent cells, at 24 h of treatment, the cells are well spread out (Figure S1C) and exhibited a typical fibroblast morphology. When the treatment time was increased to 72 h, the area of cell spreading decreased when the HDFs were exposed to sample S3 (Figure S1D). As all of the AuNPs samples contained the same amount of gold, this effect could be due to the smaller size of the AuNPs in S3.

**Figure 4:**
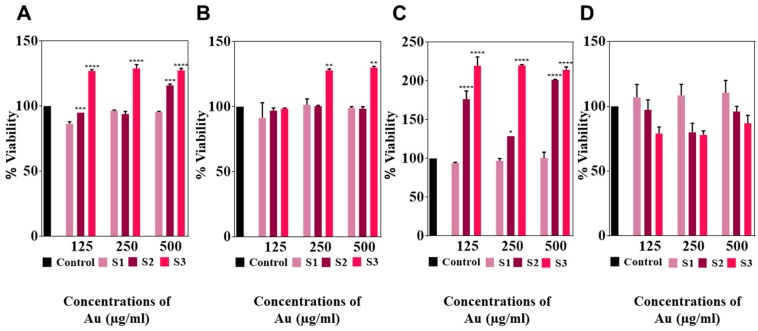
Determination of AuNPs generated cytotoxicity towards primary human dermal fibroblasts (HDFs). Cells were treated with different concentrations of AuNPs for 24 and 72 h. Cell viability of 50% confluent HDFs after 24 (**A**) and 72 h (**B**) exposure; and 80% confluent cells after 24 (**C**) and 72 h (**D**). The untreated cells served as controls. The results are represented as ±standard error mean (SEM) (*n* = 3). * *p* < 0.05, ** *p* < 0.01, *** *p* < 0.001, **** *p* < 0.0001. Asterisks indicate statistical significance compared to the control.

The viability of the HDFs in the culture conditions was determined using the resazurin assay. Figure 4A,B represents the influence of AuNPs on a 50% confluent HDFs after 24 and 72 h of treatment. The cells showed a greater than 90% viability for all of the AuNPs-HDFs treatments compared to the control. Interestingly, at both time points, the number of viable HDFs treated with S3 were significantly higher than the control, suggesting that S3 may have contributed in the proliferation of the HDFs as well.

When 80% confluent HDFs were treated with AuNPs for 24 and 72 h, the results were different (Figure 4C,D respectively). At 24 h, there was a significant increase in the viable cell number for S2 and S3 at all of the tested concentrations of Au, but S1 remained non-significant compared to the control. However, when the exposure time was increased to 72 h, a non-significant reduction in the cell viability was observed for S3. The degrees of freedom (DF) and probability (P) values determined from the viability assay for samples S1, S2, and S3 are provided in the Supporting Information (Table S1). 

It is important to note that a variable degree of cytotoxicity against a wide range of cells has been reported for gold nanoparticles [34]. However, our data indicate that none of the cacao extract derived AuNPs samples caused any acute toxicity to HDFs. In general, cacao and its phytochemical constituents are known to be beneficial for humans [22] and to promote wound healing [21,35]. In this respect, HDFs are extremely important for controlling the wound healing process [36]. The fact that there was not an adverse cytotoxic effect observed on these cells indicates that the new cacao-AuNPs have a good biocompatibility and may be useful in the field of biomedicine. The synthesized AuNPs also have potentials as nano drug carriers. The negative surface charge and the carboxyl acid groups of the oxalic acid in these AuNPs can be used to bind and deliver other antibiotics or medically relevant drugs [37].

## 4. Conclusions

Collectively, we developed a fast, single-step, and reproducible method for the synthesis of gold nanoparticles using the extract of cacao as a reducing and stabilizing agent. The resultant AuNPs were mostly spherical, had a crystalline structure, and were negatively charged. We determined the experimental conditions that lead to stable colloidal suspensions, which are important for future applications. Furthermore, the size of the nanoparticles could be tuned by adjusting the concentration of the reactants. In vitro studies suggested that the cacao derived AuNPs are biocompatible, as none of the tested formulations exhibited cytotoxicity towards 50% and 80% confluent HDFs. This is important as gold nanoparticles have gained significant attention for application in fields of medical diagnostics and therapies. The toxic chemical free method for gold nanoparticles preparation developed in this work presents also opportunities in other fields, such as sensing. The surface of the nanoparticles can potentially be functionalised with desired ligands, which will provide opportunities for surface immobilization to surfaces for various applications. Another possibility, reinforced by the tunability of nanoparticles sizes, would be attachment of drugs and biomolecules to provide vehicles for delivery of cargo inside biological cells. Overall, this exciting, simple, green, and single-step new procedure for AuNPs preparation provides endless opportunities in numerous fields of research and practical application. 

## Figures and Tables

**Table 1:** Concentrations of reactants used for the synthesis of different cacao-gold nanoparticles (AuNPs).

Sample	Gold Chloride (mg/mL)	Cacao (mg/mL)
S1	0.1	0.5
S2	0.1	1
S3	0.1	2.5
S4	0.1	10
S5	0.1	50

**Table 2:** Hydrodynamic diameter and zeta potential of green AuNPs synthesized using cacao extract.

Sample	Hydrodynamic Size (nm)	Zeta Potential (mV)
S1	54.4 ± 9.1	−11.65
S2	28.7 ± 3.4	−14.10
S3	17.9 ± 1.5	−17.52

## Supplementary Materials

The following are available online at http://www.mdpi.com/2079-4991/8/7/496/s1, Figure S1: Optical micrographs of HDFs cells exposed to different concentrations of cacao-AuNPs and the untreated controls. Panel I, panel II, and panel III denote the Au concentrations of 125, 250, and 500 µg/mL. (A) and (B) represent 50% confluent cells exposed to S1, S2, and S3 for 24 h and 72 h, respectively. HDFs of 80% confluency were incubated with AuNPs for 24 h (C) and 72 h (D).
