# Supplementary material for: “Chocolate” Gold Nanoparticles—One Pot Synthesis and Biocompatibility
Source: Nanomaterials (Basel). 2018 Jul 5;8(7):496. doi: 10.3390/nano8070496 (PMC6071287; doi:10.3390/nano8070496)
Supplement: Supplementary file 1 [file nanomaterials-08-00496-s001.pdf]

SUPPORTING INFORMATION

"Chocolate" gold nanoparticles – synthesis and applications

Neelika Roy Chowdhury [1], Allison J Cowin [2], Peter Zilm [3] and Krasimir Vasilev [1,2*]

[1] School of Engineering, University of South Australia, Mawson Lakes–SA 5095

[2] Future Industries Institute, University of South Australia, Mawson Lakes-SA 5095

[3] Microbiology laboratory, The School of Dentistry, The University of Adelaide, Adelaide-SA 5005

*Corresponding author email address: Krasimir.vasilev@unisa.edu.au

Email addresses:

neelika.roy_chowdhury@mymail.unisa.edu.au

Allison.Cowin@unisa.edu.au

peter.zilm@adelaide.edu.au

Krasimir.Vasilev@unisa.edu.au

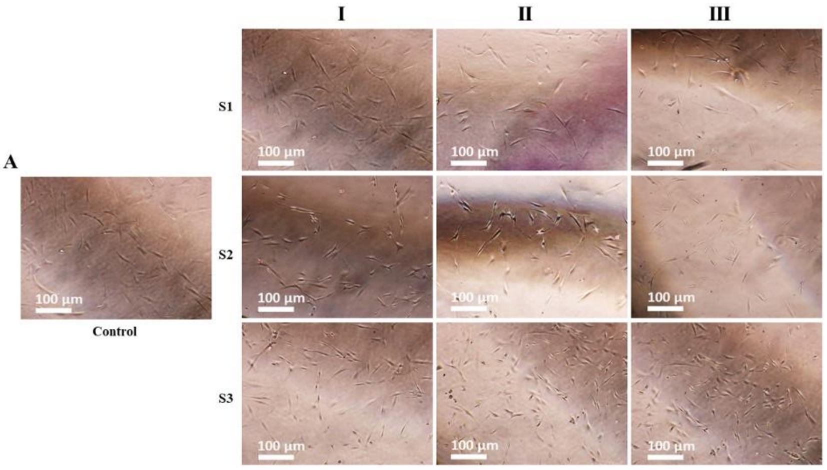

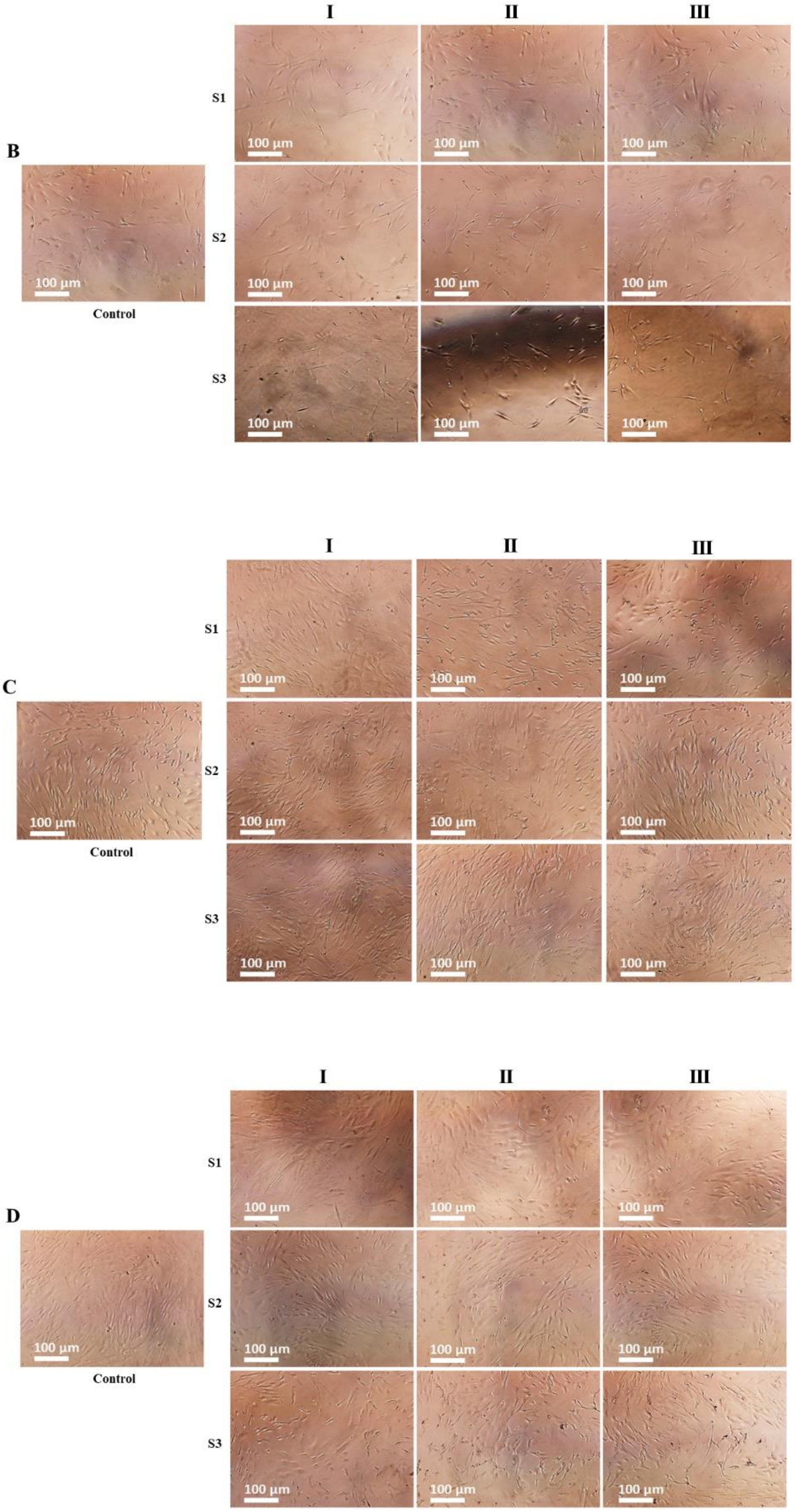

Figure S1. Optical micrographs of HDFs cells exposed to different concentrations of cacao-AuNPs and the untreated controls. Panel I, panel II and panel III denote the Au concentrations of 125, 250 and 500 µg/ml. (A) and (B) represent 50% confluent cells exposed to S1, S2 and S3 for 24 h and 72 h respectively. 80% confluent HDFs incubated with AuNPs for 24 h (C) and 72 h (D).

Table S1. DF (The degrees of freedom) and P (probability) values obtained from cell viability assay.

**Treatments and exposure time**			**DF value**	**P value**	**Adjusted P value**
50% confluent HDFs exposed for 24 h	125 µg/ml of Au	C vs S1	9 (Treatment between columns)	<0.001	0.0003
		C vs S2		ns	0.1589
		C vs S3		<0.0001	<0.0001
	250 µg/ml of Au	C vs S1	9 (Treatment between columns)	ns	0.4513
		C vs S2		ns	0.0734
		C vs S3		<0.0001	<0.0001
	500 µg/ml of Au	C vs S1	9 (Treatment between columns)	ns	0.2300
		C vs S2		<0.001	0.0001
		C vs S3		<0.0001	<0.0001
50% confluent HDFs exposed for 72 h	125 µg/ml of Au	C vs S1	9 (Treatment between columns)	ns	0.6048
		C vs S2		ns	0.9968
		C vs S3		ns	0.9996
	250 µg/ml of Au	C vs S1	9 (Treatment between columns)	ns	0.9996
		C vs S2		ns	0.9999
		C vs S3		<0.01	0.0039
	500 µg/ml of Au	C vs S1	9 (Treatment between columns)	ns	0.9997
		C vs S2		ns	0.9996
		C vs S3		<0.01	0.0024
80% confluent HDFs exposed for 24 h	125 µg/ml of Au	C vs S1	9 (Treatment between columns)	ns	0.9735
		C vs S2		<0.0001	<0.0001
		C vs S3		<0.0001	<0.0001
	250 µg/ml of Au	C vs S1	9 (Treatment between columns)	ns	0.9994
		C vs S2		<0.05	<0.0280
		C vs S3		<0.0001	<0.0001
	500 µg/ml of Au	C vs S1	9 (Treatment between columns)	ns	>0.9999
		C vs S2		<0.0001	<0.0001
		C vs S3		<0.0001	<0.0001
80% confluent HDFs exposed for 72 h	125 µg/ml of Au	C vs S1	9 (Treatment between columns)	ns	0.9759
		C vs S2		ns	0.9996
		C vs S3		ns	0.2457
	250 µg/ml of Au	C vs S1	9 (Treatment between columns)	ns	0.9348
		C vs S2		ns	0.2850
		C vs S3		ns	0.2110
	500 µg/ml of Au	C vs S1	9 (Treatment between columns)	ns	0.8420
		C vs S2		ns	0.9994
		C vs S3		ns	0.6855
